# Deuterium body array for the simultaneous measurement of hepatic and renal glucose metabolism and gastric emptying with dynamic 3D deuterium metabolic imaging at 7 T

**DOI:** 10.1002/nbm.4926

**Published:** 2023-04-02

**Authors:** Ayhan Gursan, Arjan D. Hendriks, Dimitri Welting, Pim A. de Jong, Dennis W. J. Klomp, Jeanine J. Prompers

**Affiliations:** ^1^ Center for Image Sciences University Medical Center Utrecht Utrecht The Netherlands; ^2^ Department of Radiology University Medical Center Utrecht Utrecht The Netherlands

**Keywords:** deuterium metabolic imaging, gastric emptying, glucose metabolism, kidney, liver

## Abstract

Deuterium metabolic imaging (DMI) is a novel noninvasive method to assess tissue metabolism and organ (patho)physiology in vivo using deuterated substrates, such as [6,6’‐^2^H_2_]‐glucose. The liver and kidneys play a central role in whole‐body glucose homeostasis, and in type 2 diabetes, both hepatic and renal glucose metabolism are dysregulated. Diabetes is also associated with gastric emptying abnormalities. In this study, we developed a four‐channel ^2^H transmit/receive body array coil for DMI in the human abdomen at 7 T and assessed its performance. In addition, the feasibility of simultaneously measuring gastric emptying, and hepatic and renal glucose uptake and metabolism with dynamic 3D DMI upon administration of deuterated glucose, was investigated. Simulated and measured B_1_^+^ patterns were in good agreement. The intrasession variability of the natural abundance deuterated water signal in the liver and right kidney, measured in nine healthy volunteers, was 5.6% ± 0.9% and 4.9% ± 0.7%, respectively. Dynamic 3D DMI scans with oral administration of [6,6’‐^2^H_2_]‐glucose showed similar kinetics of deuterated glucose appearance and disappearance in the liver and kidney. The measured gastric emptying half time was 80 ± 10 min, which is in good agreement with scintigraphy measurements. In conclusion, DMI with oral administration of [6,6’‐^2^H_2_]‐glucose enables simultaneous assessment of gastric emptying and liver and kidney glucose uptake and metabolism. When applied in patients with diabetes, this approach may advance our understanding of the interplay between disturbances in liver and kidney glucose uptake and metabolism and gastric emptying, at a detail that cannot be achieved by any other method.

## Graphical abstract

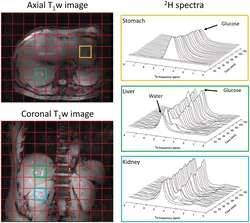

**Abbreviations used**
^8^F‐FDG [^18^F]: ‐fluoro‐2‐deoxy‐D‐glucose
AFI: actual flip angle imaging
AMARES: advanced method for accurate, robust, and efficient spectral fitting
BMI: body mass index
CV: coefficient of variation
DMI: deuterium metabolic imaging
FOV: field of view
PET: positron emission tomography
RF: radiofrequency
ROI: region of interest
SAR: specific absorption rate
S‐matrix: scattering matrix
SNR: signal‐to‐noise ratio
T_50_
: gastric emptying half time
TCA: tricarboxylic acid
VOI: volume of interest

## 1 INTRODUCTION

The maintenance of whole‐body glucose homeostasis is essential for metabolic health. In type 2 diabetes, glucose homeostasis is impaired, resulting in hyperglycemia. The liver and, more recently, also the kidneys have been recognized as playing an important role in the impaired whole‐body glucose homeostasis in type 2 diabetes.^1^, ^2^, ^3^

Under fasting conditions, only the liver and kidney can produce glucose and release it into the circulation (75%–80% normally comes from the liver, 20%–25% from the kidney).^4^ After a meal, the liver stores glucose in the form of glycogen and hepatic glucose output is suppressed in healthy humans.^1^ By contrast, renal glucose release is increased about twofold in the postprandial state.^5^ The kidneys also take up a significant amount of glucose, namely 10%–15% of all glucose utilized by the body in the postabsorptive state, and even more after meal ingestion.^5^, ^6^, ^7^ However, in contrast to the liver, glycogen content in the healthy kidney is negligible.^8^

In type 2 diabetes, both hepatic and renal glucose metabolism are dysregulated.^1^, ^2^, ^3^, ^7^ In the liver, this results in increased hepatic glucose production and impaired glycogen synthesis.^1^, ^9^ In the diabetic kidney, both glucose release and glucose uptake are significantly elevated, in postabsorptive as well as postprandial conditions.^2^, ^3^ The excessive renal glucose uptake in diabetes patients has been linked to the development of diabetic nephropathy and the accumulation of glycogen in diabetic kidneys.^10^, ^11^

In addition to the alterations in liver and renal glucose metabolism, gastric emptying abnormalities are frequently observed in diabetes and include both slow/delayed and fast gastric emptying.^12^, ^13^ While delayed gastric emptying can cause hypoglycemia in diabetes patients using exogenous insulin, rapid gastric emptying is emerging as a major determinant of postprandial hyperglycemia.^14^, ^15^, ^16^, ^17^, ^18^, ^19^ Simultaneous measurements of hepatic and renal glucose metabolism and gastric emptying would be of interest in diabetes, but are currently not feasible because of the different nature of the techniques to measure these parameters.

The current gold standard for the assessment of gastric emptying is scintigraphy, which involves radiation.^20^, ^21^ Tissue glucose metabolism is mostly studied by arterio‐venous difference measurements with both stable isotope and radioactive tracers, which is an invasive technique and, in the case of the liver, complicated by the fact that the portal vein is difficult to access. Moreover, because hepatic and renal blood flow are high, arterio‐venous difference measurements of hepatic and renal glucose metabolism are challenging.^22^ Measuring hepatic glucose metabolism quantitatively with [^18^F]2‐fluoro‐2‐deoxy‐D‐glucose (^18^F‐FDG) positron emission tomography (PET) is also complicated, as the liver receives blood from both the hepatic artery and the portal vein and deriving an image‐based input function is difficult.^23^, ^24^ Moreover, ^18^F‐FDG cannot be metabolized via glycolysis and its conversion to ^18^F‐glycogen in the liver is too slow to be detected.^25^ ^13^C magnetic resonance spectroscopy (MRS) has been used to directly measure the rate of glycogen synthesis in the liver upon mixed meal ingestion or during clamping, where part of the ingested/infused glucose is replaced by [1‐^13^C]‐glucose.^26^, ^27^ However, spatial localization is challenging for ^13^C MRS and the signal is usually acquired from a large slice selected with 1D image‐selected in vivo spectroscopy.^9^, ^28^, ^29^ Because of the low intrinsic sensitivity of ^13^C MRS, the long T_1_ relaxation times of ^13^C nuclei, and the limited field of view of commonly used ^13^C surface coils, simultaneous spatially resolved measurement of hepatic and renal glucose metabolism and gastric emptying with ^13^C MRS imaging (MRSI) is not feasible.

Deuterium metabolic imaging (DMI) is a novel technique to study metabolism in vivo.^30^, ^31^ The natural abundance of deuterium (^2^H) is very low (~0.0115%) and only ^2^H signals from deuterated water and/or lipids can be detected in vivo at natural abundance.^30^, ^32^ DMI relies on ^2^H MRSI combined with the administration of deuterated compounds, such as deuterated glucose. This allows measurement of the uptake of the deuterated compound in a specific tissue and its conversion into metabolic products as a result of its involvement in (different) metabolic pathways. In comparison with ^13^C, the T_1_ relaxation times of the quadrupolar ^2^H nuclei are about an order of magnitude shorter, allowing short repetition times and thus fast averaging in a 3D MRSI experiment.^33^, ^34^ We recently showed that the sensitivity of DMI scales supra‐linearly with the magnetic field strength and that the application of DMI at ultrahigh field leads to a significant gain in signal‐to‐noise ratio (SNR), which can be employed to reduce the scan time or to improve spatial resolution.^35^

In this study, we developed a four‐channel ^2^H transmit/receive body array coil for DMI in the human abdomen at 7 T. We characterized the coil performance and investigated the feasibility of simultaneously measuring gastric emptying, and hepatic and renal glucose uptake and metabolism with dynamic 3D DMI upon administration of deuterated glucose.

## 2 METHODS

### 2.1 Hardware

**FIGURE 1:**
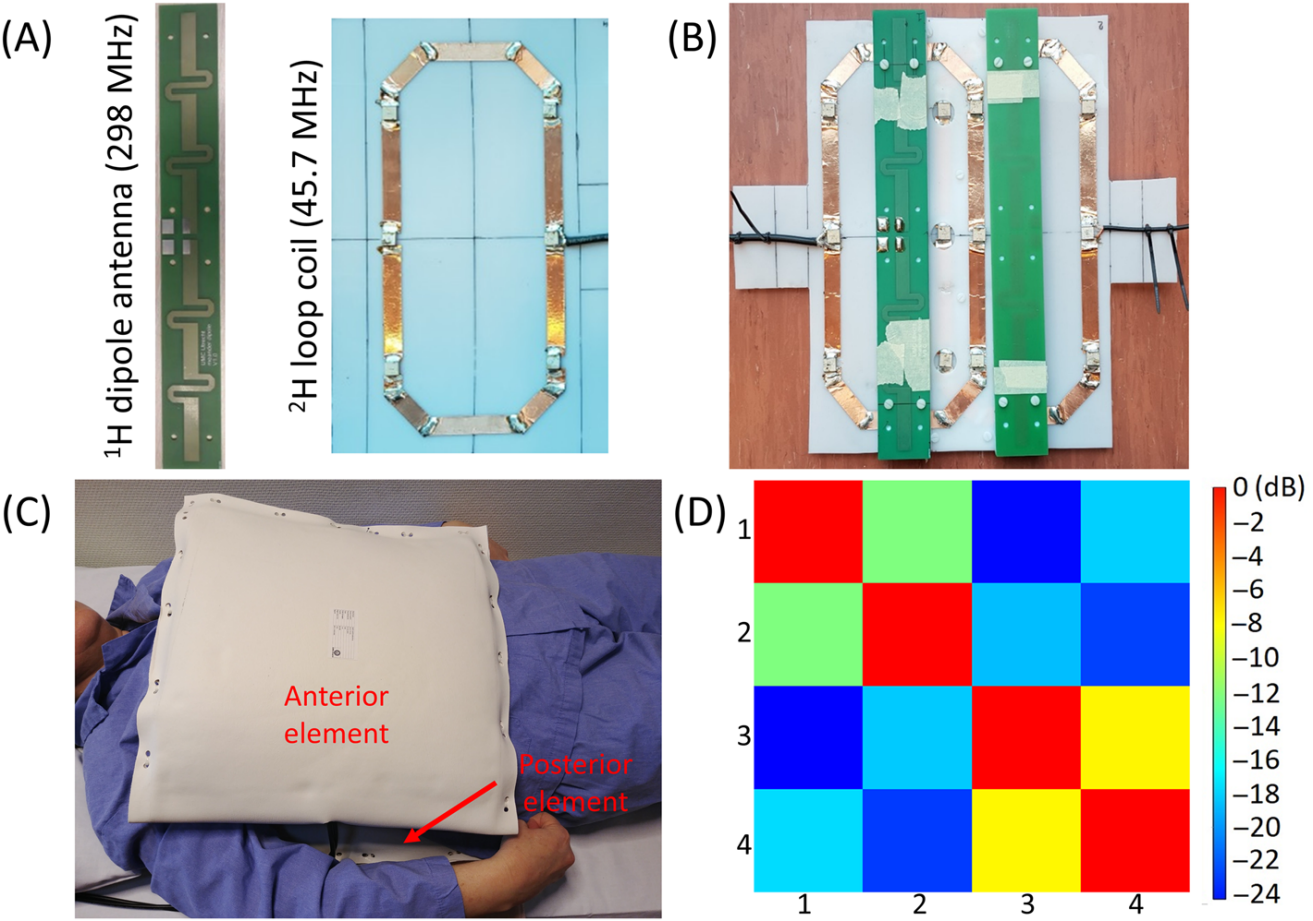
The developed four‐channel DMI body array coil. (A) ^1^H fractionated dipole antenna (length: 30.4 cm) and ^2^H transmit/receive loop (width: 12.65, length: 27.65 cm) shown separately. (B) One coil element with two ^2^H transmit/receive loops and two ^1^H fractionated dipole antennas. The two ^2^H transmit/receive loops are geometrically decoupled. (C) The coil elements are placed on anterior and posterior sides of the subject. (D) S‐matrix of the coil when loaded with a body phantom (3.5 g/l NaCl in water). Relatively strong correlations were observed for the adjacent coils within the anterior and posterior elements, but coupling between the coils of opposite elements was much lower. DMI, deuterium metabolic imaging; S‐matrix, scattering matrix.

A body array coil for DMI was developed for a 7‐T whole‐body MRI system (Philips Healthcare, Best, The Netherlands). The coil array consists of four ^2^H transmit/receive loops (width: 12.65, length: 27.65 cm) tuned at 45.7 MHz, combined with four ^1^H fractionated dipole antennas^36^ (length: 30.4 cm) tuned at 298 MHz for B_0_ shimming and anatomical imaging (Figure 1). Two ^2^H loops and ^1^H antennas were incorporated in a posterior element and two other two ^2^H loops and ^1^H antennas in an anterior element (Figure 1B,C). The ^2^H loops within each element were geometrically decoupled by overlapping them. Measurement of the scattering matrix (S‐matrix) was carried out on the bench with a body phantom (filled with water and 3.5 g/l NaCl) using a vector network analyzer (TR1300/1, Copper Mountain, Indianapolis, IN, USA). The four ^1^H fractionated dipole antennas were interfaced to 2 kW peak power amplifiers of the eight‐channel multitransmit system. The ^2^H transmit/receive loops were interfaced to a single 10 kW peak power solid state RF amplifier (AN8112, Analogic Corporation, Peabody, MA, USA). The amplifier was driven at 4 kW output and then split into the four channels, resulting in 1 kW delivered peak power for the ^2^H RF pulses to each coil. The power output of the amplifier was calibrated and the maximum local body specific absorption rate (SAR) was controlled by the coil configuration file (that specifies the SAR per unit of effective B_1_ in W/kg/μT^2^) and the reference drive scale (calibrated at 5 kW), while setting the reference B_1_ level to 38.25 μT (see Sections 2.2 and 2.3). Because ^2^H MRS is not supported in the multinuclear scanning environment of the Philips system, the ^17^O nucleus was selected and the gyromagnetic ratio was changed from 5.7718 to 6.5359 MHz/T to operate at the ^2^H frequency. Furthermore, a four‐channel receiver board of a 1‐T system was used to convert the ^2^H analogue signal to a digital signal.

### 2.2 Electromagnetic simulations

Sensitivity and RF safety of the body array coil were evaluated with electromagnetic (EM) simulations. Finite‐difference time‐domain simulations (Sim4Life, ZMT, Zurich, Switzerland) were performed at 45.7 MHz on the multitissue model Duke (IT'IS Foundation, Zurich, Switzerland). ^2^H loops were placed on the right side of the body of Duke, matching the center of the loops to the height of the liver. For simplicity, ^1^H antennas were omitted in the EM simulations, as coupling between the ^2^H loops and ^1^H dipole antennas is negligible.^37^ In the simulations, the loop elements were made of perfect electric conductive material, tuned to 45.7 MHz, matched to 50 Ω and decoupled by overlapping adjacent elements as in our design. The B_1_^+^ field and SAR over 10 g tissue (SAR_10g_) was simulated in linear mode for 1 W total input power over all ^2^H loops. The maximum SAR_10g_ per unit of power was used to calculate the SAR settings in the coil configuration file (see Section 2.3).

### 2.3 B_1_^+^ mapping in a phantom

^2^H B_1_^+^ maps were acquired on a body size phantom containing 3 g/l NaCl, 43 mM KH_2_PO_4_, and 0.54% D_2_O, using the actual flip angle imaging (AFI) method^38^ (3D gradient echo, TE = 2.5 ms, TR_1_/TR_2_ = 50/400 ms, field of view [FOV] = 240 × 480 × 300 mm^3^, resolution = [10 × 10 × 20] mm^3^, pulse shape = block, nominal flip angle = 60°, reference B_1_^+^ = 38.25 μT). Measured actual flip angle values were converted to B_1_^+^/√P, where P is the RF input power (4 kW), using the reference B_1_^+^ of 38.25 μT. The power level of 4 kW to provide a presumed reference B_1_^+^ of 38.25 μT was fixed and not modified for subjects. Consequently, the maximum SAR per unit of power was converted to maximum SAR per unit of B_1_ field and stored in the coil configuration file, to ensure that the SAR will not exceed 10 W/kg.

### 2.4 In vivo signal acquisition

The study was approved by the institutional medical ethics committee and written informed consent was obtained from the volunteers. The anterior and posterior coils were positioned on the right side of the body to maximize the acquired signals from the liver and right kidney. Before the DMI experiments, conventional MRI was performed to optimize the B_0_ field (B_0_ shimming) and to make anatomical reference images for DMI planning. First, T_1_‐weighted (T_1_w) gradient echo localizer images were acquired. Then low‐flip‐angle gradient echo images (2D multislice acquisition, TE = 1.68 ms, TR = 30 ms, FOV = 350 × 457 × 30 mm^3^, in‐plane resolution = [3.9 × 3.8] mm^2^, slice thickness = 10 mm, flip angle = 3.5°) were acquired in three slices for every transmit channel, and were used for RF phase shimming.^39^ Optimum phase settings were calculated, with equal amplitude for each channel, to obtain maximum signal intensity in a region of interest (ROI) (averaged over three slices) by numerical minimization in MATLAB (MathWorks, Natick, MA, USA). A 3D B_0_ map (3D gradient echo, TE = 1.49 ms, ΔTE = 1.0 ms, TR = 10 ms, FOV = 280 × 402 × 78 mm^3^, resolution = [4.4 × 6.0 × 6.0] mm^3^, flip angle = 5°) was acquired for B_0_ shimming. This scan was acquired during a breath‐hold in the exhaled state. Linear and second order shim settings were optimized using MRCode software (TeslaDC, Zaltbommel, The Netherlands).^40^ Finally, axial and coronal T_1_w images and Dixon^41^ anatomical reference images were acquired with the same FOV and the same number of slices as for the DMI scans.

All DMI measurements were performed with a 3D MRSI sequence^42^ using a block pulse for excitation and Hamming‐weighted k‐space sampling^43^ (Figure S1). Deuterium signals were collected with a 5 kHz spectral bandwidth and 1024 datapoints. The TR was 333 ms for all scans and no respiratory gating was applied.

### 2.5 Deuterium pulse length optimization

To determine the optimal pulse length of the deuterium block pulse for the used TR of 333 ms, a series of DMI scans with low spatial resolution (nominal voxel size = 30 × 30 × 30 mm^3^, FOV = 240 [anterior–posterior {AP}] × 300 [right–left {RL}] × 270 [foot–head {FH}] mm^3^, number of signal averages [NSA] = 4 in the center of k‐space, and an acquisition time of 5 min 8 s per scan) was performed with block pulse lengths of 0.5, 0.67, 0.83, and 1.0 ms in two healthy volunteers at natural abundance (one male and one female). In the center of the liver, the highest deuterated water signal intensity was obtained with a 1‐ms block pulse (Figure S2). Therefore, for the rest of the study, 1‐ms block pulses were used, resulting in an effective TE of 1.95 ms.

### 2.6 Assessment of intrasession deuterium signal stability

The stability of the in vivo DMI measurements was assessed in nine volunteers at natural abundance. In six volunteers, five dynamic DMI scans were acquired with a nominal voxel size of 30 × 30 × 30 mm^3^ (effective voxel size = 109 ml), FOV = 240 (AP) × 300 (RL) × 270 (FH) mm^3^, NSA = 4 in the center of k‐space, and an acquisition time of 5 min 8 s per scan. In the three other volunteers, five dynamic DMI scans were acquired with a nominal voxel size of 25 × 25 × 25 mm^3^ (effective voxel size = 63 ml), FOV = 250 (AP) × 300 (RL) × 300 (FH) mm^3^, NSA = 4 in the center of k‐space, and an acquisition time of 10 min 35 s per scan.

### 2.7 Dynamic DMI with deuterated glucose administration

One healthy volunteer (female, 49 years of age) was scanned twice, about 3 months apart, after an overnight fast. [6,6’‐^2^H_2_]‐glucose (0.75 g per kg body weight; Buchem B.V., Apeldoorn, The Netherlands) was dissolved in ~300 ml of water and administered orally. During the first session, the deuterated glucose was administered via a tube while the volunteer was in the scanner, after the acquisition of two baseline DMI scans at natural abundance. DMI scans were continued up to 130 min after glucose intake. A high temporal resolution was used in this experiment, to monitor the initial response to deuterated glucose intake. The DMI acquisition parameters were nominal voxel size = 30 × 30 × 30 mm^3^, FOV = 240 (AP) × 300 (RL) × 270 (FH) mm^3^, NSA = 4 in the center of k‐space, and an acquisition time of 5 min 8 s per scan.

During the second session, the deuterated glucose was administered 30 min before the volunteer went into the scanner. The volunteer waited sedentarily on a chair before going into the scanner. The acquisition of DMI scans was started 68 min after glucose intake and the scans were continued up to 205 min after intake. In this session, the spatial resolution was increased, at the cost of a lower temporal resolution: nominal voxel size 25 × 25 × 25 mm^3^, FOV = 250 (AP) × 300 (RL) × 300 (FH) mm^3^, NSA = 4 in the center of k‐space, and an acquisition time of 10 min 35 s per scan.

### 2.8 Signal reconstruction, processing, and analysis

All the reconstruction and processing steps were performed with an in‐house written MATLAB script. To obtain a Hamming‐weighted k‐space the acquisitions were added in k‐space. After that, an additional correction on the weighting coefficients for k‐space locations was applied to better match the discrete number of acquired averages with the ideal Hamming function (Figure S1C). Fourier transformation was then performed in the spatial domains, followed by phase correction. Signals from the four channels were combined using the Roemer equal noise algorithm.^44^ SNR was calculated as the water peak height divided by the standard deviation of the noise (20–30 ppm spectral region) in the frequency domain spectra. For visualization purposes, spectra were apodized with a 5‐Hz exponential function and zero‐filled to 2048 points.

For each voxel, deuterated water (4.7 ppm), glucose (3.8 ppm; only for scans with deuterated glucose), and lipid (1.3 ppm) signals were fitted in the time domain with AMARES^45^, ^46^ using pure Lorentzian line shapes.

For the dynamic scans acquired at natural abundance, the intrasession stability of the deuterated water signal was determined by calculating the coefficient of variation (CV), that is, the standard deviation of the water amplitude divided by the mean water amplitude for the five dynamic scans, for all voxels within volumes of interest (VOIs) covering the whole liver and the right kidney, respectively.

For the dynamic scans acquired with deuterated glucose administration, fitted water and glucose amplitudes in the liver and the kidney were normalized to the water amplitude of the first time point in the same voxel and glucose amplitudes were corrected for the number of deuterium nuclei. For visualization of the glucose signal in the liver and kidney, first, ROIs were drawn on axial and coronal T_1_w images. Color maps were then generated using normalized glucose amplitudes within the ROIs, which were overlaid on axial and coronal T_1_w images. To assess the kinetics of glucose uptake and metabolism in the liver and kidney, normalized water and glucose amplitudes were averaged for the voxels within VOIs in the liver and kidney, respectively.

### 2.9 Calculation of the gastric emptying rate

The gastric emptying rate was determined from the DMI experiments performed after deuterated glucose administration inside the scanner. Axial T_1_w images were used to draw VOIs for the proximal and distal parts of the stomach and the sum of the two VOIs was defined as the total stomach VOI (Figure S3). Only the deuterated glucose signal was fitted with AMARES within proximal and distal VOIs and color maps were generated from non‐normalized glucose amplitudes. To create time curves, glucose signal amplitudes were added within each VOI. The glucose signal in the first measurement after glucose intake in the total stomach VOI was set to 100%. For the total stomach VOI, the time course of glucose signal amplitudes was fitted to a monoexponential function to determine the gastric emptying half time (T_50_).

### 2.10 Statistical analysis

Statistical analysis was performed using Student's paired *t*‐test or Pearson's correlation (MATLAB R2022a, MathWorks). A *p* value of less than 0.05 was considered significant.

## 3 RESULTS

The S‐matrix obtained for the ^2^H loop coils, measured with the load of a body phantom, is shown in Figure 1D. Relatively strong correlations were observed for the adjacent coils within the anterior and posterior elements, that is, –12 and −9 dB, respectively. Coupling between the coils of opposite elements was much lower with a mean value of −21 ± 2.6 dB.

**FIGURE 2:**
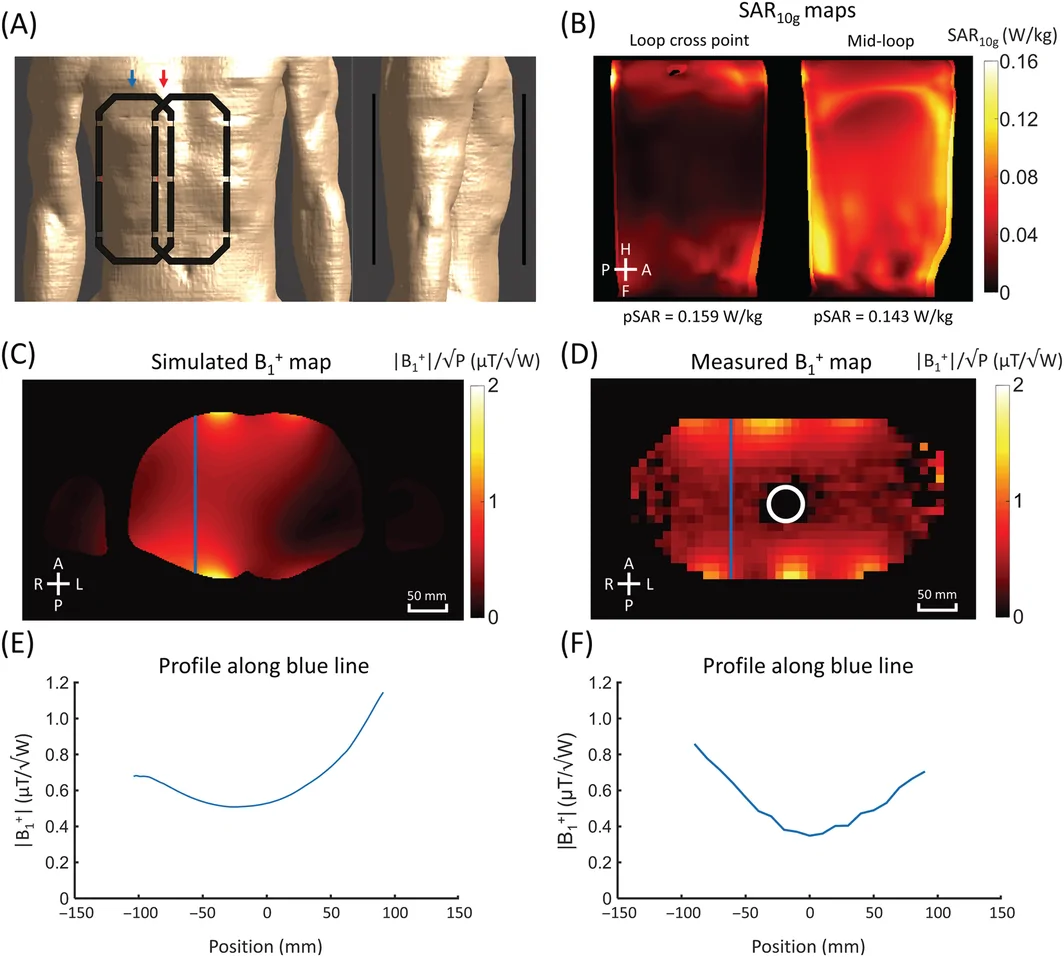
Results of electromagnetic (EM) simulations and phantom measurements with the four‐channel DMI body array coil. (A) Coil positioning on the Duke model in EM simulations shown in coronal and sagittal view. (B) Sagittal slices of the SAR_10g_ map at the cross point of the loops (red arrow in (A)) and in the center of the lateral loops (blue arrow in (A)). Peak local SAR_10g_ (pSAR) with 1 W input power was 0.159 W/kg at the cross point of the loops. (C) Simulated axial B_1_^+^ map (|B_1_^+^|/√P) at the center of the liver. (D) Measured axial B_1_^+^ map on a D_2_O‐enriched (0.54%) body size phantom, normalized to input power (B_1_^+^/√P), for comparison with EM simulations. The phantom has a cylindrical hole (filled with air), which is indicated by the white circle. (E) and (F) Traces (in the AP direction) of the simulated and measured B_1_^+^ maps in (C) and (D), respectively. The positions of the traces are indicated by the blue lines in panels (C) and (D). AP, anterior–posterior; DMI, deuterium metabolic imaging; SAR, specific absorption rate.

Simulated SAR_10g_ and B_1_^+^ maps for 1 W input power are shown in Figure 2. Local SAR_10g_ peaked at a value of 0.159 W/kg at the closest point of the body to the superior crossing point of the loops in the posterior element (Figure 2A,B). In the sagittal slice at the midpoint of lateral loop coils, SAR_10g_ had a maximum value of 0.143 W/kg at the closest point of the Duke model to the coil elements (Figure 2A,B). The simulated B_1_^+^ maps (|B_1_^+^|/√P) showed higher B_1_^+^ amplitudes in the proximity of the loop coils at both the anterior and posterior sides, and lower amplitudes in the center of the body (Figure 2C,E). The experimentally determined B_1_^+^/√P maps, measured on a body size phantom enriched with D_2_O, showed similar patterns, but with 1.5‐fold lower amplitudes (Figure 2D,F).

**FIGURE 3:**
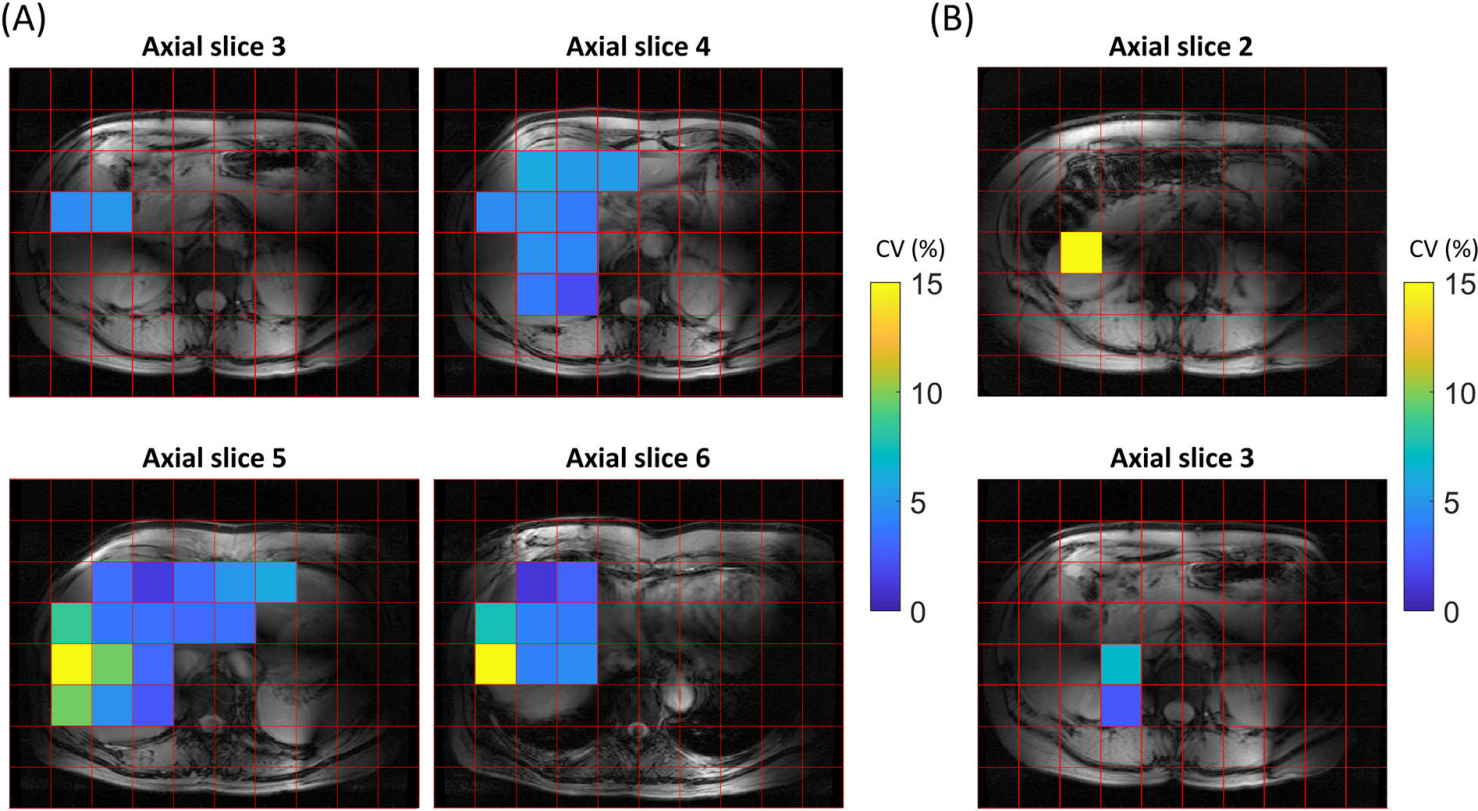
Intrasession coefficient of variation (CV) for the natural abundance deuterated water signal in the (A) Liver and (B) Kidney for dynamic DMI scans in one of the volunteers (volunteer 3 in Table 1). Axial slices of T_1_w images overlaid with the DMI grid (voxel size = 30 × 30 × 30 mm^3^) and intrasession CV values for voxels within the drawn VOIs in the (A) Liver and (B) Right kidney. For the whole liver and kidney VOIs, the average CV was 6.1% ± 6.9% and 4.7% ± 3.3%, respectively. DMI, deuterium metabolic imaging; T_1_w, T_1_‐weighted; VOI, volume of interest.

The stability of the natural abundance deuterated water signal amplitude in repeated in vivo DMI scans (without deuterated glucose administration) was assessed in nine healthy volunteers using two different voxel sizes. Table 1 summarizes the results averaged over the liver and right kidney VOIs in all volunteers and Figure 3 shows spatially resolved liver CVs in one volunteer. SNR and linewidths of the water signal and CVs of the water signal amplitude were similar among the different volunteers and also for both spatial resolutions (Table 1). SNR, linewidth, or CV did not significantly correlate with body mass index (BMI). However, SNR correlated negatively with the body circumference of the subjects (measured at the height of the liver) for both liver (R = –0.73, *p* < 0.05) and kidney (R = –0.72, *p* < 0.05), and in the liver the CV was positively correlated with the circumference (R = 0.76, *p* < 0.05). SNR tended to be higher (*p* = 0.07) and the CV and linewidth tended to be lower (*p* = 0.06 and *p* = 0.07, respectively) in the right kidney compared with the liver. The mean intrasession CV for all subjects was 5.6% ± 0.9% for the liver and 4.9% ± 0.7% for the right kidney.

**TABLE 1:** Results of repeated in vivo DMI scans at natural abundance: intrasession CV, SNR, and linewidth of the natural abundance deuterated water signal.

Volunteer	BMI (kg/m^2^)	Circumference at liver height (cm)	Nominal voxel size	No. voxels		CV (%)		SNR		Linewidth (Hz)	
				Liver	Kidney	Liver	Kidney	Liver	Kidney	Liver	Kidney
1	21.8	86.2	27 ml	38	3	5.1 ± 2.5	4.3 ± 2.9	12.5 ± 3.5	16.8 ± 1.4	28.6 ± 4.9	21.3 ± 2.9
2	23.3	86.1	27 ml	44	2	5.6 ± 3.3	5.9 ± 2.4	13.2 ± 4.8	17.7 ± 1.9	27.8 ± 5.3	22.9 ± 3.5
3	24.7	84.3	27 ml	37	3	6.1 ± 6.9	4.7 ± 3.3	15.4 ± 6.2	18.7 ± 0.1	24.5 ± 5.4	19.4 ± 3.0
4	25.3	90.2	27 ml	53	3	6.3 ± 5.1	5.8 ± 1.6	14.8 ± 4.1	13.1 ± 3.9	26.5 ± 4.7	28.2 ± 4.1
5	26.3	99.7	27 ml	71	4	6.8 ± 5.5	5.3 ± 1.1	10.0 ± 4.3	7.1 ± 3.2	27.6 ± 5.1	31.3 ± 3.7
6	33.8	99.8	27 ml	49	4	6.5 ± 3.8	4.0 ± 1.3	12.3 ± 4.2	16.6 ± 3.9	25.6 ± 4.5	19.3 ± 2.0
7	24.7	81.6	15.6 ml	54	6	4.1 ± 2.2	5.0 ± 2.1	15.7 ± 4.3	19.4 ± 4.6	24.5 ± 4.8	22.9 ± 5.1
8	23.3	88.6	15.6 ml	57	4	4.9 ± 2.1	4.8 ± 0.4	14.4 ± 4.3	17.2 ± 2.6	28.0 ± 6.0	22.1 ± 2.7
9	21.8	88.7	15.6 ml	65	5	5.3 ± 2.7	4.2 ± 2.0	15.9 ± 4.8	15.6 ± 2.7	24.9 ± 4.9	25.7 ± 2.7
Mean						5.6 ± 0.9	4.9 ± 0.7	13.8 ± 2.0	15.8 ± 3.7	26.4 ± 1.6	23.7 ± 4.0

*Note*: Data are given as means ± SD for the voxels within the VOI drawn on the liver and on the right kidney. Abbreviations: BMI, body mass index; CV, coefficient of variation; DMI, deuterium metabolic imaging; SNR, signal‐to‐noise ratio; VOI, volume of interest.

**FIGURE 4:**
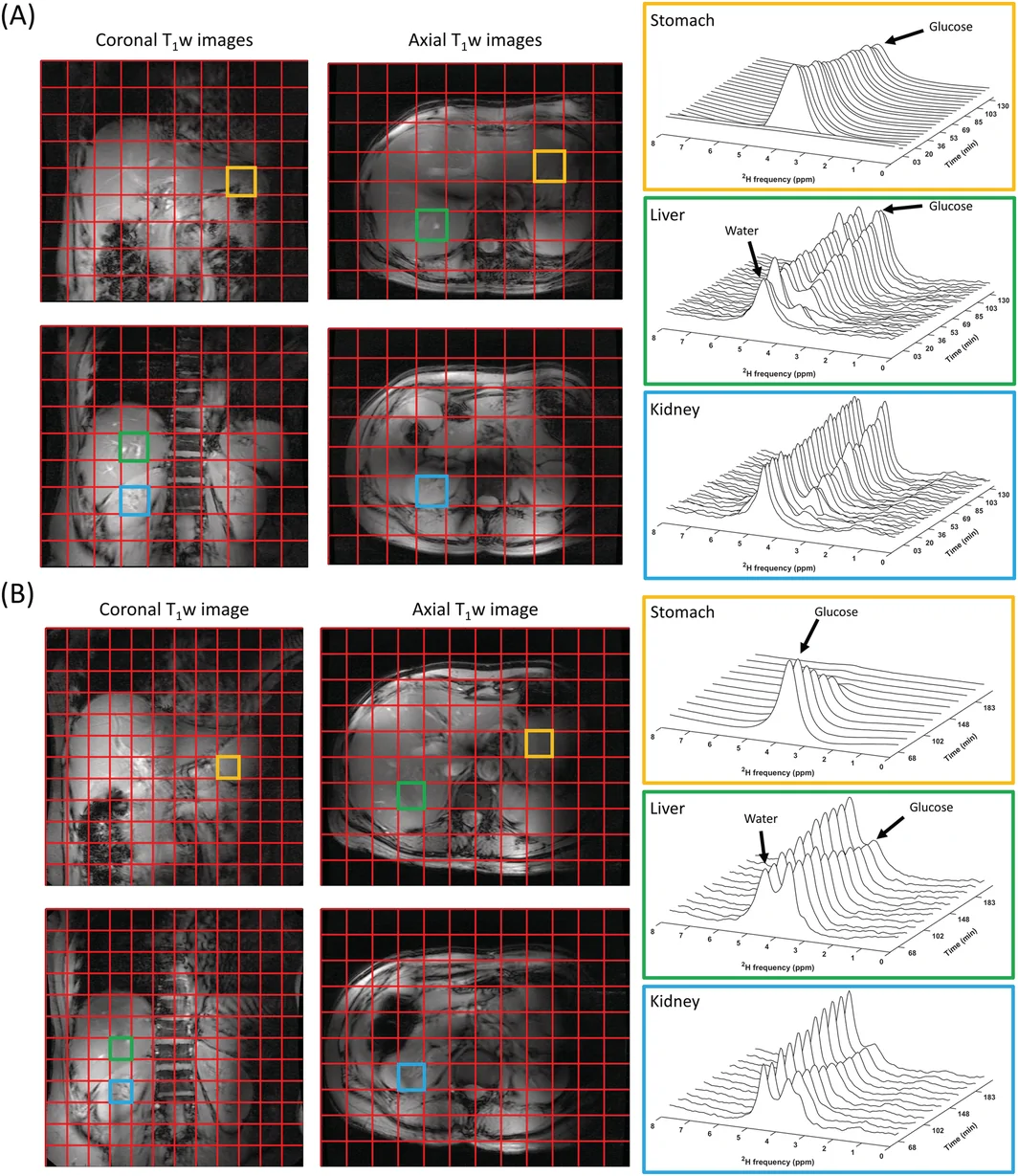
(A) Dynamic 3D DMI measurements at baseline and 0–130 min after oral administration of [6,6’‐^2^H_2_]‐glucose (first dataset) and (B) 68–205 min after oral administration of [6,6’‐^2^H_2_]‐glucose (second dataset). Left: DMI grid overlaid on axial and coronal T_1_w images. Right: deuterium spectra over time from a voxel located in the stomach (orange), the liver (green), and the right kidney (blue). In the first dataset, a very strong glucose signal was observed in the stomach immediately after intake, which slowly decayed thereafter. By contrast, in voxels located in the liver and kidney, the deuterated glucose and (to a lesser extent) water signal increased over time. In the second dataset, a substantial amount of deuterated glucose was still present in the stomach in the first dynamic, but it had mostly disappeared at the end of the experiment. In the liver and kidney, the deuterated glucose signal gradually decayed, while the deuterated water signal kept increasing. DMI, deuterium metabolic imaging; T_1_w, T_1_‐weighted.

Raw (spectral) data from the DMI scans with deuterated glucose administration are shown in Figure 4. In the dataset collected from baseline up to 130 min after deuterated glucose intake, a very strong glucose signal was observed in the stomach immediately after intake, which slowly decayed thereafter (Figure 4A). By contrast, in voxels located in the liver and kidney, the deuterated glucose signal increased over time, together with a slower increase of the deuterated water signal (Figure 4A). In the DMI data obtained from 68 until 205 min after glucose intake (second dataset), a substantial amount of deuterated glucose was still present in the stomach in the first dynamic, but it had mostly disappeared at the end of the experiment (Figure 4B). In the liver and kidney, the deuterated glucose signal gradually decayed, while the deuterated water signal kept increasing (Figure 4B).

**FIGURE 5:**
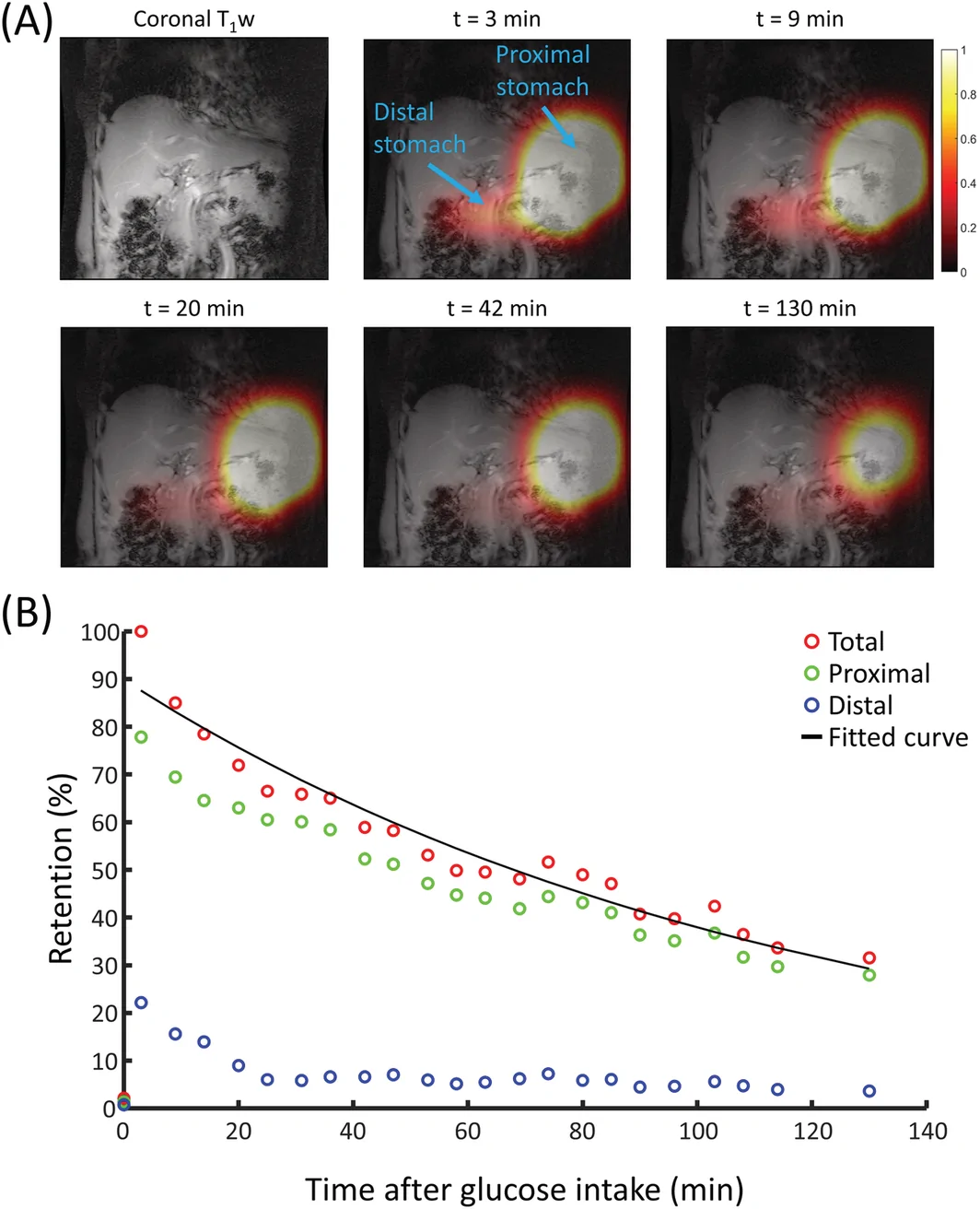
(A) Coronal color maps of fitted deuterated glucose signal amplitudes (in AU) from five selected 3D DMI measurements between 0 and 130 min after oral administration of [6,6’‐^2^H_2_]‐glucose. Signal amplitudes larger than 1 are represented in white. The color maps are overlaid on the T_1_w images. (B) Glucose retention graphs for total, proximal, and distal stomach, together with a monoexponential fit of the total stomach signal. Glucose retention is defined as the sum of the deuterated glucose signal amplitudes within each VOI, expressed as a percentage of the glucose signal in the total stomach VOI in the first measurement after deuterated glucose intake (= 100%). About 80% of the glucose signal was initially observed in the proximal stomach, which then decreased more or less linearly over time. In the distal stomach, the glucose signal decayed rapidly during the first 20 min and then remained at a constant low value (~7%) up to 130 min. The gastric emptying half time (T_50_) for the total stomach determined from the fit was 80 ± 10 min. DMI, deuterium metabolic imaging; T_1_w, T_1_‐weighted; VOI, volume of interest.

The spatial distribution and retention of the deuterated glucose signal in the stomach from 0 to 130 min after intake of deuterated glucose was analyzed in more detail in Figure 5. About 80% of the glucose signal was initially observed in the proximal stomach, and then decreased more or less linearly over time. In the distal stomach, the glucose signal decayed rapidly during the first 20 min and then remained at a constant low value (~7%) up to 130 min. For the total stomach glucose signal, the T_50_ obtained from a monoexponential fit was 80 ± 10 min.

**FIGURE 6:**
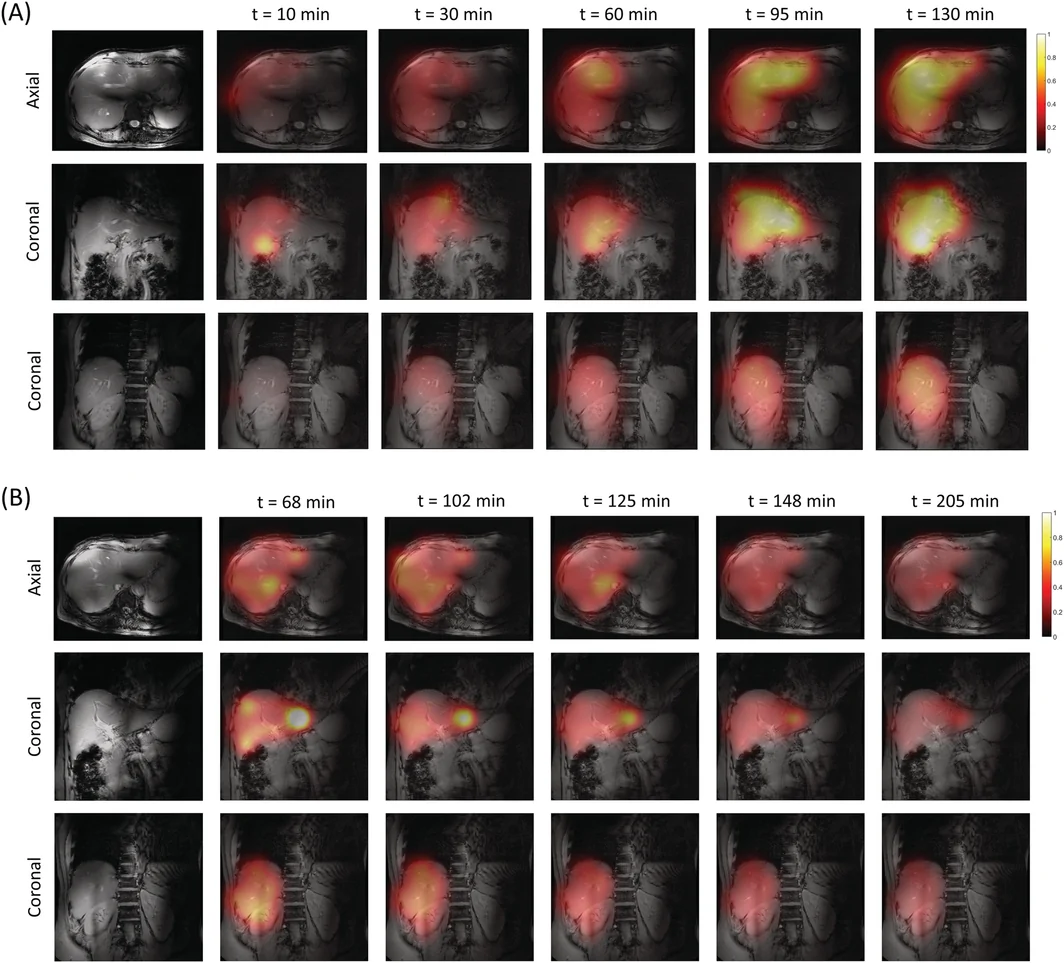
Axial and coronal color maps of normalized deuterated glucose amplitudes in the liver and right kidney for five selected 3D DMI measurements between (A) 0 and 130 min and (B) 68 and 205 min after oral administration of [6,6’‐^2^H_2_]‐glucose. Axial T_1_w images were used to draw ROIs on the liver and the right kidney. For each voxel within the ROIs, fitted glucose amplitudes were normalized to the deuterated water amplitude of the first time point in the same voxel, and these normalized glucose amplitudes within the ROIs were used to generate the color maps. The same scale was used for all color maps and color maps are overlaid on the anatomical axial and coronal T_1_w images. In (A), the deuterated glucose signal keeps increasing over time, whereas in (B) it gradually decreases in both the liver and right kidney. DMI, deuterium metabolic imaging; ROI, region of interest; T_1_w, T_1_‐weighted.

Normalized deuterated glucose maps generated for the liver and right kidney for different time points between 10 and 130 min after deuterated glucose intake (first dataset) and between 68 and 205 min after intake (second dataset) are shown in Figure 6. The contrast was very similar in the liver and kidney throughout both datasets. Fitted water and glucose amplitudes in the liver and right kidney were also averaged over VOIs and analyzed as a function of time (Figure S4). The kinetics of deuterated glucose increase and subsequent decrease were very similar in the liver and kidney. Also, the slow increase in deuterated water signal was similar in both tissues. However, because of signal bleeding from the very intense deuterated glucose signal from the stomach, which was shifted to ~4.5 ppm because of B_0_ inhomogeneities, the water signal amplitude was likely overestimated for some of the first time points, which is particularly obvious for the first time point after glucose intake in both liver and kidney (Figure S4).

## 4 DISCUSSION

In this study, we developed a four‐channel deuterium body array for DMI at 7 T and characterized its performance. Using this setup, we demonstrated the feasibility of monitoring glucose uptake and metabolism in two organs, which play an important role in whole‐body glucose homeostasis, that is, the liver and the kidney, upon oral administration of deuterated glucose, simultaneously with the measurement of gastric emptying.

EM simulations showed that the maximum local SAR_10g_ is 0.159 W/kg per 1 W of total power (Figure 2B). The simulated B_1_^+^ patterns were very similar to the measured B_1_^+^ patterns, but the experimental B_1_^+^ amplitudes were 66% of the simulated amplitudes (Figure 2C–F). The lower experimental B_1_^+^ amplitudes can be explained by RF power losses in the cables, power splitter, coil conductors, and T/R switches. Including these losses in the SAR_10g_ calculations, the maximum SAR_10g_ value with a 1 ms block pulse at 4 kW peak power and a TR of 333 ms would be 0.83 W/kg (0.159 × [0.66]^2^ × 4000 × 1/333), which is far below the SAR_10g_ limits for the human body (restricted by the MR system to 10 W/kg). Care must be taken that losses can also be obtained due to destructive B_1_^+^ interference, when phase settings between channels are incorrectly set. The SAR per unit of power may not be substantially affected, but the SAR per unit of B_1_ can be dramatically affected. Consequently, we used a fixed power level per unit of assumed B_1_ to ensure safe use of the setup. In fact, when excluding all potential losses, the SAR would be 1.91 W/kg, that is, still much lower than the 10 W/kg set by the MR system or the 20 W/kg set by the International Electro‐technical Comission (IEC) guidelines.

The sensitivity of the ^2^H MRSI measurements with the four‐channel ^2^H transmit/receive loop coil array was high enough to detect natural abundance deuterated water and lipid signals in vivo (Figure S2). Preclinical studies showed that after administration of deuterated glucose, the signal from deuterated glucose in the liver reaches a similar or even higher amplitude as/than the natural abundance deuterated water signal, depending on the route of administration.^47^ Considering that the natural abundance deuterated water signal amplitude is in the relevant range for DMI measurements with deuterated glucose and that, without deuterated substrate administration, the deuterated water signal should be constant, measurements for calibration and the assessment of variability can conveniently be done at natural abundance using this signal. The intrasession variability of the ^2^H MRSI measurements determines the sensitivity to detect changes in metabolite signals during dynamic DMI scans. The intrasession variability (CV) of the natural abundance deuterated water signal in a VOI covering the whole liver, measured in nine healthy volunteers with two different spatial resolutions, was 5.6% ± 0.9% (Table 1), which is considerably lower than the metabolite changes expected in dynamic scans after administration of deuterated glucose. In volunteers with a larger body circumference, the voxels in the center of the liver had a lower SNR, resulting in slightly higher average liver CVs. In the right kidney, the SNR tended to be higher and the linewidth tended to be lower than in the liver, resulting in a CV of 4.9% ± 0.7%, which also tended to be lower than in the liver. In addition to measurement errors, physiological motion (in particular, respiratory motion) likely contributed to the observed variations, as the ^2^H MRSI measurements were performed during free breathing. Implementation of motion correction could reduce the variability by improving data quality, for example, by decreasing linewidths, and by correcting ghosting caused by motion.^48^, ^49^, ^50^ However, even without motion correction, the variability of free‐breathing liver and kidney ^2^H MRSI data was excellent and sufficient for the dynamic measurement of uptake and metabolism of deuterated glucose.

Administration of deuterated glucose inside the scanner allowed measurement of gastric emptying of glucose (Figure 5). Gastric emptying is a highly regulated process, ensuring that the amount of calories entering the duodenum is limited and fairly constant.^12^, ^14^, ^51^ As a result, the gastric emptying rate after intake of a glucose drink is affected by the glucose load, as has been shown using scintigraphy.^52^, ^53^ Although the intragastric distribution of a glucose drink is different between sitting and lying positions, both the overall gastric emptying rate and glycemic response are not significantly different for both positions.^54^ Therefore, we can still compare our DMI measurements of overall gastric emptying with scintigraphy measurements, which are usually performed in sitting position, as long as the glucose load is similar. Compared with a scintigraphy study in sitting healthy subjects using a similar glucose load (50 g of glucose) as in our study, we observed a slightly shorter T_50_ for the whole stomach (80 ± 10 vs. 103 ± 12 min).^13^ The intragastric distribution of the glucose load and the different kinetics for proximal and distal gastric emptying (Figure 5B) agree well with scintigraphy results of subjects in a lying position.^54^ Although scintigraphy is easy to operate and noninvasive, it comes with a radiation burden for the subject.^55^ As gastric emptying is emerging as a major determinant of postprandial glycemia,^14^, ^15^, ^16^, ^17^, ^18^, ^19^ its measurement is very relevant, especially in the context of obesity and type 2 diabetes. We have shown here that DMI can provide a sensitive, radiation‐free alternative to scintigraphy for the assessment of gastric emptying.

In the liver DMI spectra, a peak emerged at 3.8 ppm right after administration of deuterated glucose (Figure 4A) and this signal increased up to the end of the first experiment. In the second experiment, the signal at 3.8 ppm was gradually decaying (Figure 4B). While signals from both [6,6’‐^2^H_2_]‐glucose and [6,6’‐^2^H_2_]‐glycogen resonate at ~3.8 ppm, the observed signal most likely originates solely from deuterated glucose, as it was shown that detection of deuterated glycogen is not feasible in vivo, due to the very short T_2_ relaxation time.^47^ It has been estimated that after oral intake of a glucose load, ~25%–40% of the glucose (which enters the liver through the portal vein) is taken up by hepatocytes.^27^, ^56^ However, hepatic blood volume is as much as 25–30 ml/100 g liver weight and therefore part of the signal could also originate from blood,^57^ which is a limitation of the method. The decrease of the deuterated glucose signal in the liver at later time points is (apart from a decrease in blood concentration) most likely explained by its conversion into (^2^H MRS invisible) glycogen and possibly hepatic glucose release.^58^ At the same time, we observed a small but gradual increase in deuterated water signal throughout the first and second experiments, which could originate from water labeling during glycolysis and the tricarboxylic acid (TCA) cycle. However, the deuterated water is not necessarily produced in the liver itself, but likely originates (also) from other tissues.^59^ This would be in agreement with the fact that signals from deuterated lactate or glutamate were not detectable in the liver.

In the kidney DMI data, both the observed spectral patterns and the kinetics of deuterated glucose appearance and disappearance were similar to those in the liver DMI data (Figures 4 and S4). However, healthy kidneys contain negligible amounts of glycogen.^3^, ^4^ Thus, in contrast to the liver, the disappearance of the deuterated glucose signal in the kidney cannot be explained by the conversion of glucose into glycogen.

In this study, we investigated the feasibility of DMI to assess glucose uptake and metabolism in the human liver and kidney. Like the liver, the kidneys also contain high volumes of blood in their vasculature network (cortical blood volume: ~40 ml/100 g kidney weight^60^). Our study protocol did not include blood sampling for the analysis of blood glucose deuterium enrichments, which would be required to quantitatively separate tissue and blood contributions to the deuterated glucose signals in the DMI data. The high volume and concentration of deuterated glucose in the stomach distorted the deuterium spectra in adjacent voxels during the first ~30 min after oral intake. This problem of signal bleeding could be solved by increasing the spatial resolution, at the cost of a lower temporal resolution. Given the SNR, which was more than sufficient to detect deuterated glucose, acceleration techniques could be implemented to achieve both a high spatial and temporal resolution.^61^ Although deuterated glucose signal amplitudes were normalized with respect to the deuterated water signal, this does not perfectly correct for the coil sensitivity profile, due to the effect of B_1_^+^ inhomogeneities in combination with T_1_ relaxation time differences between deuterated water and deuterated glucose. However, this does not affect the observed kinetics of deuterated glucose appearance and disappearance at a specific location.

## 5 CONCLUSION

The development of a four‐channel deuterium body array coil enabled dynamic DMI in the human abdomen with a FOV covering the liver, kidney, and stomach. Upon oral administration of [6,6’‐^2^H_2_]‐glucose, liver and kidney glucose uptake and metabolism could be determined, together with the assessment of gastric emptying. When applied in patients with type 2 diabetes, this approach may advance our understanding of the interplay between disturbances in liver and kidney glucose metabolism and gastric emptying, at a detail which cannot be achieved by any other method.

## Supporting information


**Figure S1.** (A) 3D magnetic resonance spectroscopic imaging (MRSI) sequence diagram. (B) Hamming weighting acquisition pattern, with 4 averages in the center of the k‐space and a minimum of one acquisition in the outer regions of k‐space. (C) Point spread function (PSF) without (blue) and with (orange) Hamming weighting during acquisition. With additional correction of the k‐space weighting coefficients during reconstruction, data are apodized with an ideal, Hamming function (black).
**Figure S2.** Deuterium flip angle optimization in vivo. Center axial slice of in vivo 3D DMI scans (voxel size = 30 × 30 × 30 mm^3^, TR = 333 ms) acquired with a block pulse of 0.5 ms (A, C) and 1.0 ms (B, D) overlaid on Dixon water (A, B) and fat (C, D) images. (E, F) Deuterated water signal intensity as a function of pulse length for voxels in the blue and orange rectangles in panel C. The direction is indicated by the arrows and in both panel E and F the most left graph is from the voxel at the surface (anterior in panel E and posterior in panel F) and the most right graph from the center. At the surface, the highest deuterated water signal intensity was obtained with a 0.5 ms block pulse, but in the center of the liver, the highest signal was obtained with a 1 ms block pulse.
**Figure S3.** VOI's for distal (blue) and proximal (green) parts of the stomach. Masks were drawn on T_1_w axial images. The VOI for the distal part of the stomach was drawn in the axial slice in which both kidneys were visible.
**Figure S4.** Fitted deuterated water and glucose peak amplitudes from the liver and right kidney (averaged over VOI's) as a function of time (A) from baseline up to 130 min after deuterated glucose intake and (B) from 68 until 205 min after deuterated glucose intake (B). For both liver and kidney, glucose and water amplitudes were referenced to the water amplitude at the first time point of the respective dataset. The glucose signal amplitude was corrected for the number of contributing deuterium nuclei. Because of signal bleeding from the very intense deuterated glucose signal from the stomach, which was shifted to ~4.5 ppm due to B_0_ inhomogeneities, the water signal amplitude was likely overestimated for the first time points in panel (A) for both liver and kidney.
