# Supplementary material for: Deuterium body array for the simultaneous measurement of hepatic and renal glucose metabolism and gastric emptying with dynamic 3D deuterium metabolic imaging at 7 T
Source: NMR Biomed. 2023 Apr 2;36(8):e4926. doi: 10.1002/nbm.4926 (PMC13587257; doi:10.1002/nbm.4926)
Supplement: Supplementary file 1 [file NBM-36-e4926-s001.docx]

Supporting Information

Deuterium body array for the simultaneous measurement of hepatic and renal glucose metabolism and gastric emptying with dynamic 3D deuterium metabolic imaging at 7T

Ayhan Gursan^1^, Arjan D. Hendriks^1^, Dimitri Welting^1^, Pim A. de Jong^2^, Dennis W.J. Klomp^1^, Jeanine J. Prompers^1^

*^1^Center for Image Sciences, University Medical Center Utrecht, Utrecht, The Netherlands*

*^2^Department of Radiology, University Medical Center Utrecht, Utrecht, The Netherlands*

**Figure S1.** (A) 3D magnetic resonance spectroscopic imaging (MRSI) sequence diagram. (B) Hamming weighting acquisition pattern, with 4 averages in the center of the k-space and a minimum of one acquisition in the outer regions of k-space. (C) Point spread function (PSF) without (blue) and with (orange) Hamming weighting during acquisition. With additional correction of the k-space weighting coefficients during reconstruction, data are apodized with an ideal, Hamming function (black).

**Figure S2.** Deuterium flip angle optimization *in vivo*. Center axial slice of *in vivo* 3D DMI scans (voxel size = 30 × 30 × 30 mm^3^, TR = 333 ms) acquired with a block pulse of 0.5 ms (A, C) and 1.0 ms (B, D) overlaid on Dixon water (A, B) and fat (C, D) images. (E, F) Deuterated water signal intensity as a function of pulse length for voxels in the blue and orange rectangles in panel C. The direction is indicated by the arrows and in both panel E and F the most left graph is from the voxel at the surface (anterior in panel E and posterior in panel F) and the most right graph from the center. At the surface, the highest deuterated water signal intensity was obtained with a 0.5 ms block pulse, but in the center of the liver, the highest signal was obtained with a 1 ms block pulse.

**Figure S3.** VOI’s for distal (blue) and proximal (green) parts of the stomach. Masks were drawn on T_1_w axial images. The VOI for the distal part of the stomach was drawn in the axial slice in which both kidneys were visible.

**Figure S4.** Fitted deuterated water and glucose peak amplitudes from the liver and right kidney (averaged over VOI’s) as a function of time (A) from baseline up to 130 min after deuterated glucose intake and (B) from 68 until 205 min after deuterated glucose intake (B). For both liver and kidney, glucose and water amplitudes were referenced to the water amplitude at the first time point of the respective dataset. The glucose signal amplitude was corrected for the number of contributing deuterium nuclei. Because of signal bleeding from the very intense deuterated glucose signal from the stomach, which was shifted to ~4.5 ppm due to B_0_ inhomogeneities, the water signal amplitude was likely overestimated for the first time points in panel (A) for both liver and kidney.
